# Effect of Intramammary Dry Cow Antimicrobial Treatment on Fresh Cow’s Milk Microbiota in California Commercial Dairies

**DOI:** 10.3390/antibiotics11070963

**Published:** 2022-07-18

**Authors:** Carl Basbas, Sharif Aly, Emmanuel Okello, Betsy M. Karle, Terry Lehenbauer, Deniece Williams, Erika Ganda, Martin Wiedmann, Richard V. Pereira

**Affiliations:** 1 Department of Population Health and Reproduction, School of Veterinary Medicine, University of California, Davis, Davis, CA 95616, USA; ctbasbas@ucdavis.edu (C.B.); saly@ucdavis.edu (S.A.); eokello@ucdavis.edu (E.O.); tlehenbauer@ucdavis.edu (T.L.); 2 Veterinary Medicine Teaching and Research Center, University of California, Davis, Tulare, CA 93274, USA; dvmwilliams@ucdavis.edu; 3 Cooperative Extension, Division of Agriculture and Natural Resources, University of California, Orland, CA 95963, USA; bmkarle@ucanr.edu; 4 Department of Food Science, Cornell University, Ithaca, NY 14850, USA; ganda@psu.edu (E.G.); martin.wiedmann@cornell.edu (M.W.); 5 Penn State College of Agricultural Sciences, University Park, PA 16802, USA

**Keywords:** milk, mastitis, antibiotics, dry-off

## Abstract

This study used 16S rRNA sequencing to evaluate the effects of dry cow antimicrobial therapy on the udder milk microbiota by comparing the microbial populations in milk at dry-off (DRY) (~60 days before calving) and post-partum (FRESH) (4–11 days after calving) from cows receiving an intramammary antibiotic infusion prior to dry-off (IMT) and cows that did not receive treatment (CTL). Milk was collected from 23 cows from the IMT group and 27 cows from the CTL group. IMT and DRY samples had a greater correlation with the genera *Brevibacterium* and *Amaricoccus*, and the family Micrococcaceae, when compared to IMT and FRESH samples. CTL group samples collected at DRY had a greater correlation with the genera *Akkermansia* and *Syntrophus,* when compared to FRESH samples; no bacterial taxa were observed to have a significant correlation with FRESH samples in the CTL group. DRY samples collected from the CTL group had a greater correlation with the genus *Mogibacterium* when compared to IMT and CTL samples. For DRY samples collected from the IMT group, a greater correlation with the genus *Alkalibacterium* when compared to DRY and CTL samples, was observed. The lack of a correlation for FRESH samples between the CTL and IMT treatment groups indicated that intramammary antimicrobial dry cow therapy had no significant effect on the udder milk microbiota post-partum.

## 1. Introduction

Mastitis, characterized by abnormal milk, is inflammation of the mammary gland, which is responsible for significant economic loses. The great majority of mastitis cases are of bacterial origin, being one of the most prevalent infections in dairy cows, which results in the use of antimicrobial drugs [1,2]. Aside from its effects on the mammary gland, mastitis has a detrimental effect on the welfare, reproduction, and productivity of dairy cows [3,4]. A common practice at dairy farms for treating existing or chronic cases of mastitis, or preventing new mastitis cases during the dry period is the wide use of intramammary antibiotics at the time of dry-off. In 2014, 93% of all U.S. dairy cows received intramammary antimicrobials at dry-off, with 80.3% of all dairy operations choosing to use intramammary antimicrobials non-selectively on all cows at dry-off [5]. This non-selective administration of intramammary antimicrobials at dry-off, also known as blanket therapy, blanket dry cow therapy, or total dry cow therapy, is thought to account for a sizeable portion of the 15,645 kg of medically important antibiotics administered intramammary annually [6]. The antimicrobial drugs most commonly used during the dry-off period in the U.S. are the first-generation cephalosporin cephapirin (58% of all operations and 32% of all cows) and the third-generation cephalosporin ceftiofur (28% of all operations and 22% of all cows) [5]. Cephalosporin drugs, especially third generation, have been listed on the World Health Organization’s list of critically important antimicrobials for human medicine due to their role in treating important infections caused by Gram negative bacteria [7]. Therefore, identifying alternatives to reduce the need for the therapeutic use of third-generation cephalosporin drugs, especially for the prevention of infection, has become an important area for efforts in livestock medicine. 

For decades, the National Mastitis Council has recommended that all quarters of all cows be treated with intramammary antimicrobials at dry-off [8]. The rationale being that blanket therapy is more effective in preventing new infections and does not require any type of screening procedure, as compared to selective therapy. Researchers have continued to debate the effectiveness of blanket treatment, and the introduction and use of a commercial non-antibiotic internal teat sealant has only added to this discussion [9,10,11,12]. However, decreased cost is a reason some producers consider the use of selective antibiotic treatment over blanket treatment at the time of dry-off. A mathematical model from a 2007 study suggested the choice between blanket or selective treatment is highly farm specific, while a more recent model concluded that selective treatment was more economically beneficial [13,14]. Furthermore, one study has proposed the use of an algorithm to selectively treat only those cows at high risk of developing new cases of mastitis, and by using this approach, they estimated a reduction in dry cow antibiotic use by approximately 60% without any adverse effects on animal health [15]. 

A concern with intramammary antimicrobial dry cow therapy is the potential for disruption of the endogenous microbiota present in the bovine mammary gland. Additionally, there is a risk of the inadvertent introduction of environmental organisms into the udder due to contamination during the administration process of the intramammary antimicrobials. Advances in culture-independent methods of microbial analysis of the mammary gland, often via milk samples, has challenged the idea that this environment is sterile [16,17]. A recent longitudinal cohort study noted that, while both Chao richness and Shannon diversity were greater in healthy compared to inflamed mammary glands, a low sequencing success rate suggests that the milk microbiota may not be especially abundant [18,19]. Nevertheless, it is broadly accepted that a healthy microbiome contributes positively to host health, and conversely, any disruption of the microbiota may detrimentally impact the host [20]. Therefore, treatment with intramammary antimicrobial dry cow therapy could potentially decrease colonization resistance against pathogens of the mammary gland [21,22]. This has led researchers to debate whether mastitis, traditionally viewed as a host–pathogen interaction, may actually be a result of dysbiosis of the mammary microbiota [23]. 

Given the high prevalence and cost of mastitis in the dairy industry and the controversy of blanket intramammary antimicrobial dry cow therapy, more research is needed to fully understand the impacts and benefits of this practice to the microbiota. To address this aim, a subset of a repository of milk samples and its respective data from a state-wide dry cow therapy trial were employed [24]. We proposed to evaluate the effect of dry cow antimicrobial therapy on the udder milk microbiota by comparing the microbial populations in milk at dry-off (~60 days before calving) and post-partum from cows receiving an intramammary antibiotic infusion and cows that did not receive therapy.

DNA was extracted using a standardized approach [25]. Due to the small quantities of DNA present in each sample, an additional step was required to amplify DNA to a necessary concentration for 16s sequencing [26,27,28]. Taxonomy was assigned using Greengenes version 13.8 at a 99% match [29,30].

## 2. Results

### 2.1. Descriptive Data and Microbial Diversity Data

**Figure 1:**
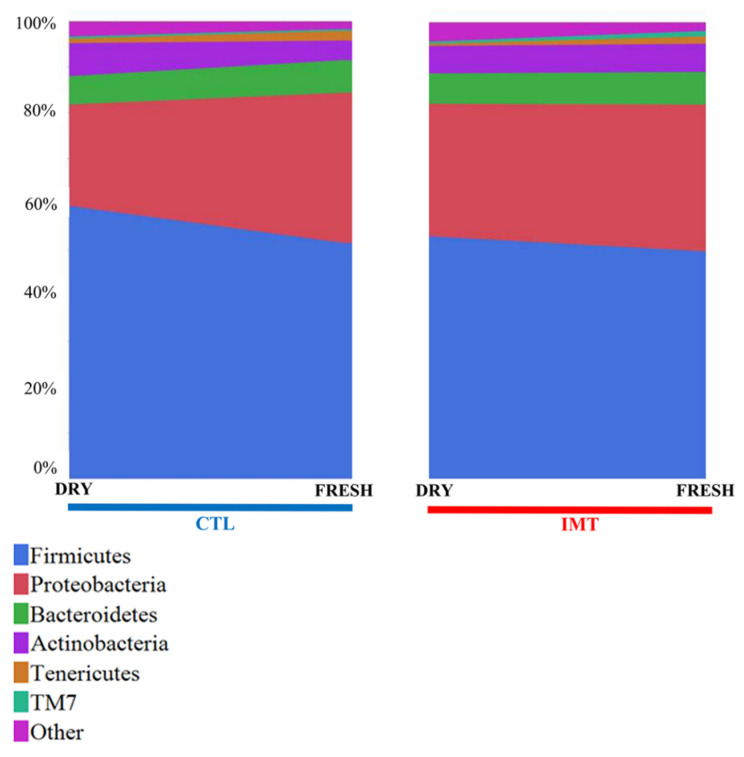
Mean percentage distribution for the top five most prevalent phyla for each sampling point by treatment group. IMT: treatment group representing cows receiving intramammary antimicrobial drug treatment at dry-off; DRY: samples collected at the time of dry-off; FRESH samples: samples collected from post-partum cows between 4 and 11 days in milk; CTL: control group representing cows not receiving intramammary antimicrobial drug treatment at dry-off.

A descriptive analysis of the 16s data revealed the top five most abundant phyla. In order, these include: Firmicutes, Proteobacteria, Bacteroidetes, Actineobacteria, and Tenericutes (Figure 1). For DRY samples, the mean values for the phyla were as follows: Firmicutes (56.6%), Proteobacteria (25.3%), Bacteroidetes (6.4%), Actineobacteria (6.6%), and Tenericutes (0.7%). For FRESH samples, the mean values for the phyla were as follows: Firmicutes (50.7%), Proteobacteria (32.5%), Bacteroidetes (7.1%), Actineobacteria (5.1%), and Tenericutes (1.9%).

**Figure 2:**
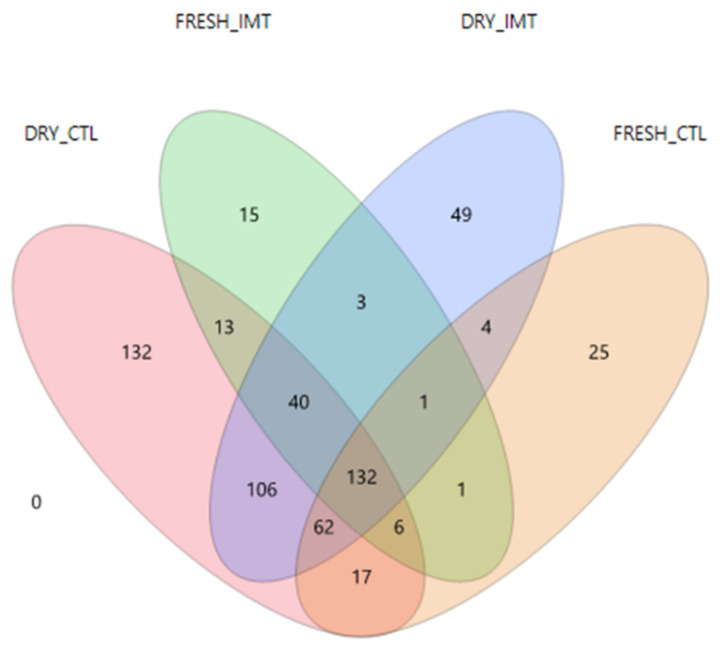
Venn diagram of the amplicon sequence variants found in milk samples from cows receiving intramammary antimicrobial treatment at dry-off (DRY and IMT), cows not receiving IMT at dry-off (DRY and CTL), and the follow up samples for these cows when fresh (FRESH and IMT, and FRESH and CTL, respectively). IMT: treatment group representing cows receiving intramammary antimicrobial drug treatment at dry-off; DRY: samples collected at the time of dry-off; FRESH: samples collected from post-partum cows between 4 and 11 days in milk; CTL: control group representing cows not receiving intramammary antimicrobial drug treatment at dry-off.

A Venn Diagram of ASVs from treatment groups for each time point displaying ASVs shared between groups is presented in Figure 2. A total of 106 ASVs were shared between DRY and CTL, and DRY and IMT, while only one ASV was shared between FRESH and CTL, and FRESH and IMT. A total of 17 ASVs were shared between DRY and CTL, and FRESH and CTL, while three ASVs were shared between DRY and IMT, and FRESH and IMT. A total of 132 ASVs were shared among all four treatment and time point combinations.

No significant differences were found in the mean relative abundance for the six genera commonly associated with mastitis (*Staphylococcus* sp., *Bacillus* sp., *Streptococcus* sp., *Mycoplasma* sp., *Escherichia* sp., and *Trueperella* sp.) for IMT and CTL, at DRY and FRESH (Supplemental Figure S1).

Relative mean abundances for the top 20 taxa ranging from the order to genus level were tabulated to identify differences in relative abundance among the four experimental groups (Table 1). The genus *Staphylococcus* was the most abundant taxa for DRY and CTL, and FRESH and IMT. The genus *Delftia* was the most abundant taxa for DRY and IMT, and FRESH and CTL.

**Figure 3:**
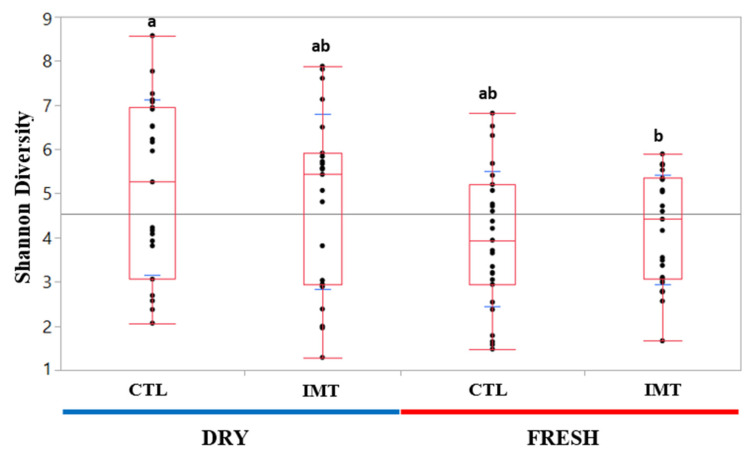
Shannon diversity quantile box plots for each treatment group for each sampling time point. Data were not normally distributed (Shapiro–Wilk *p* value = 0.044). Different letters (a and b) indicated treatment groups that had significantly different values based on Wilcoxon Sum Rank Test (*p* value < 0.05). Fresh: samples collected from post-partum cows between 4 and 11 days in milk; CTL: control group representing cows not receiving intramammary antimicrobial drug treatment at dry-off; IMT: treatment group representing cows receiving intramammary antimicrobial drug treatment at dry-off; DRY: samples collected at the time of dry-off.

Quantile box plots were generated to illustrate changes in Shannon diversity between DRY and FRESH time points for the IMT and CTL groups (Figure 3). A Wilcoxon sum rank test was performed to assess the significance of differences between experimental groups. The only Shannon diversity values deemed significantly different (*p* value < 0.05) were those from DRY and CTL, and FRESH and IMT cows.

### 2.2. Canonical Coefficients and Linear Regression

**Figure 4:**
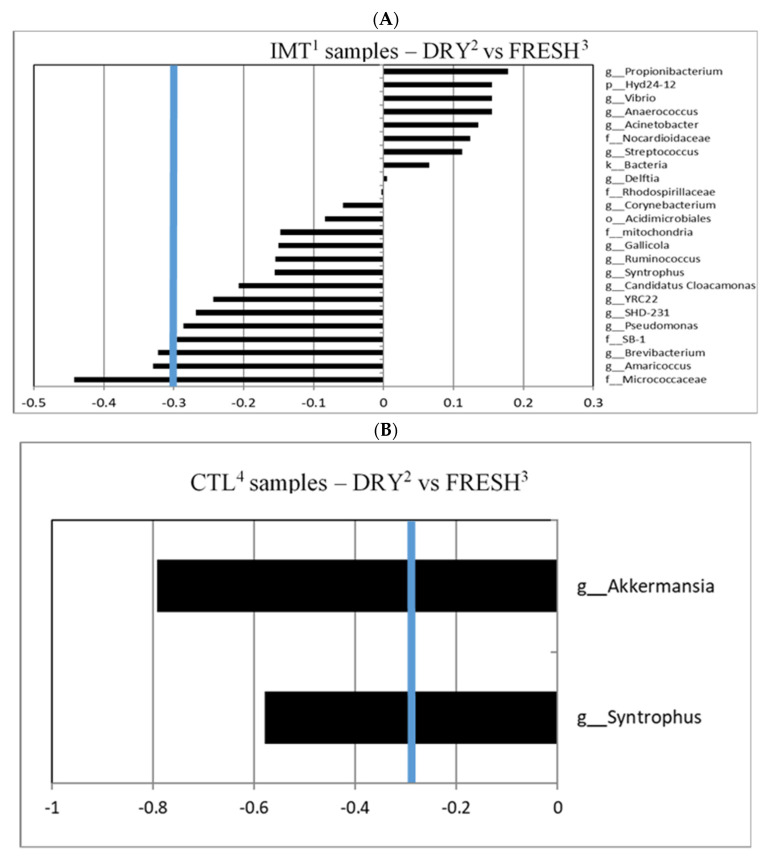
Canonical structure coefficients comparing sampling microbiota for milk collection sampling points (DRY and FRESH) by treatment group, namely cows either receiving intramammary antimicrobial treatment (IMT) at dry-off and cows not receiving IMT at dry-off (CTL). (**A**) Correlation between microbial taxa and the discriminant function for DRY vs. FRESH sampling points of IMT cows. Bacterial taxa with canonical structure coefficients ≤−0.3 or ≥0.3 (blue line) are considered important when distinguishing sampling times (DRY vs. FRESH) from IMT and CTL cows. (**B**) Correlation between microbial taxa and the discriminant function for DRY vs. FRESH sampling points of CTL cows. ^1^ IMT: treatment group representing cows receiving intramammary antimicrobial drug treatment at dry-off; ^2^ DRY: samples collected at the time of dry-off; ^3^ FRESH: samples collected from post-partum cows between 4 and 11 days in milk; ^4^ CTL: control group representing cows not receiving intramammary antimicrobial drug treatment at dry-off.

A canonical cut-off loading value of ±0.3 was used to identify taxa for different treatment groups and time points within those groups, as previously reported [31,32,33]. For the canonical analysis comparing time points by treatment, we observed that IMT group samples collected at dry-off had a greater correlation with the genera *Brevibacterium* and *Amaricoccus*, and the family Micrococcaceae, when compared to FRESH samples; no samples were indicated to have a greater correlation with FRESH samples (Figure 4A). We observed that the CTL group samples collected at DRY had a greater correlation with the genera *Akkermansia* and *Syntrophus,* when compared to FRESH samples. No samples were observed to have a significant correlation with FRESH samples in the CTL treatment group (Figure 4B).

**Figure 5:**
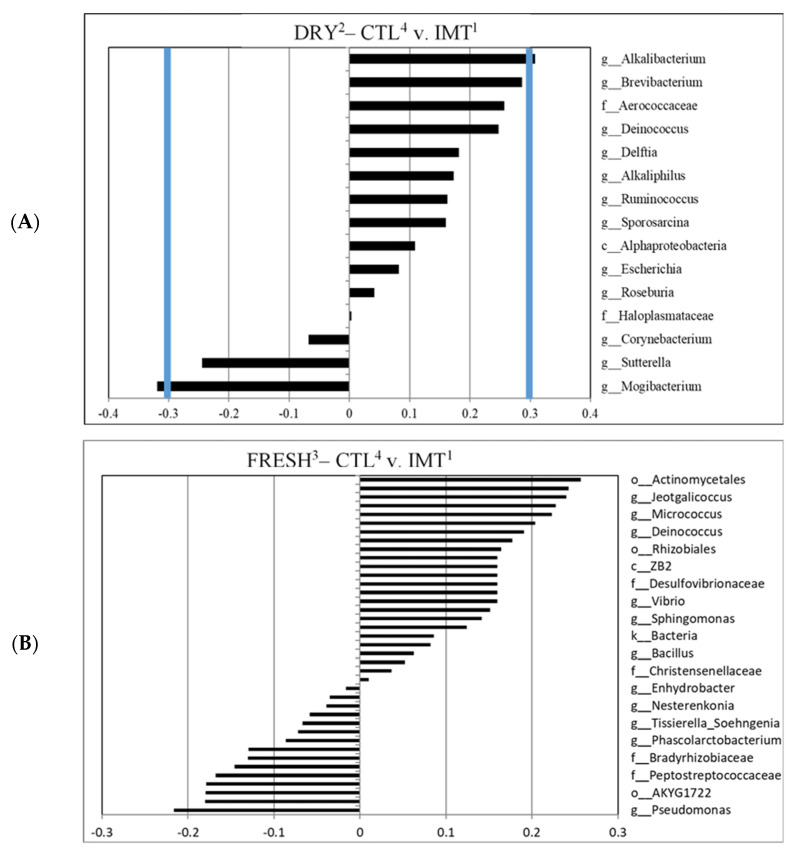
Canonical structure coefficients comparing sampling microbiota for cows receiving intramammary antimicrobial treatment (IMT) at dry−off and cows not receiving intramammary antimicrobial treatment (CTL) at dry-off. (**A**) Correlation between microbial taxa and the discriminant function for DRY, comparing CTL versus IMT samples. (**B**) Correlation between microbial taxa and the discriminant function for FRESH, comparing CTL versus IMT samples. Bacterial taxa with canonical structure coefficients ≤−0.3 or ≥0.3 (blue lines) are considered important when distinguishing between CTL and IMT samples. ^1^ IMT: treatment group representing cows receiving intramammary antimicrobial drug treatment at dry-off. ^2^ DRY: samples collected at the time of dry-off. ^3^ FRESH: samples collected from post-partum cows between 4 and 11 days in milk. ^4^ CTL: control group representing cows not receiving intramammary antimicrobial drug treatment at dry-off.

For the canonical analysis comparing treatment by time points, we observed that DRY samples collected from CTL group had a greater correlation with the genus *Mogibacterium* when compared to IMT samples. For DRY samples collected from the IMT group, a greater correlation with the genus *Alkalibacterium* when compared to CTL samples, was observed (Figure 5A). No correlations were observed for FRESH samples comparing the CTL and IMT treatment groups (Figure 5B).

No significant difference in the relative abundance of bacteria was observed between treatment by time points or between time points for different treatment groups, for taxon identified as having a significant effect on the microbial composition (canonical ± 0.3), except for *Brevibacterium* (Supplemental Table S1). Bacteria taxa evaluated included f_Micrococcaceae, g_Amaricoccus, g_Brevibacterium, g_Akkermansia, g_Syntrophus, g_Alkalibacterium, and g_Mogibacterium. Further analysis using the Tukey pairwise analysis for the genus *Brevibacterium* revealed no significant difference between the different treatment groups by time points (Supplemental Table S2).

**Figure 6:**
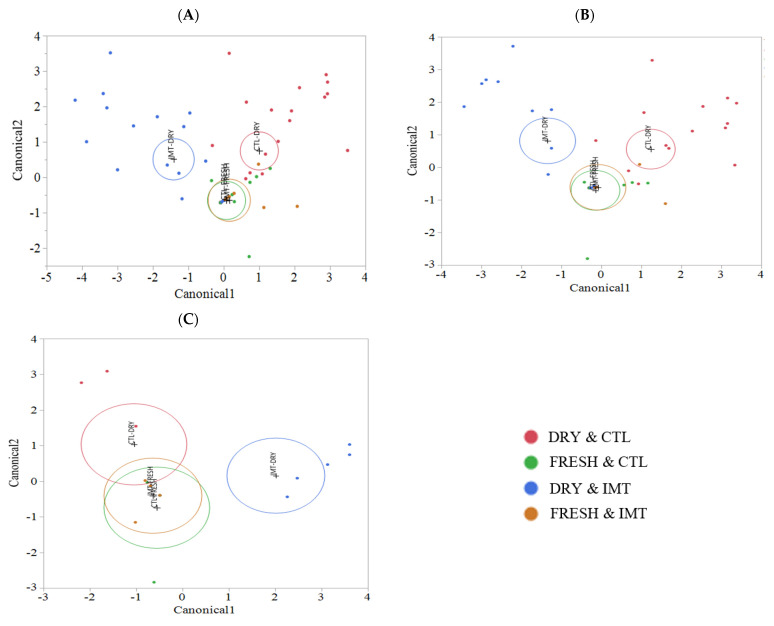
Discriminant analyses of milk sample microbiomes for each treatment group by sampling point for IMT cows treated with either: (**A**) cephapirin and cloxacillin (**B**) cephapirin only (**C**) cloxacillin only. Bacterial relative abundance was used as covariates, and treatment group as the categorical variable (NR = red dots, DR = blue dots). An ellipse indicates the 95% confidence region to contain the true mean of the group, and a plus symbol indicates the center (centroid) of each group. IMT: treatment group representing cows receiving intramammary antimicrobial drug treatment at dry−off; DRY: samples collected at the time of dry−off; FRESH samples: samples collected from post-partum cows between 4 and 11 days in milk; CTL: control group representing cows not receiving intramammary antimicrobial drug treatment at dry-off.

Linear discriminant analysis of milk samples for each treatment group by time points is displayed in Figure 6. This analysis was stratified by the antimicrobial drug used for the IMT infusion (cephapirin vs. cloxacillin) and is also displayed (Figure 6B,C, respectively). Samples from FRESH, regardless of whether collected from the IMT or CTL cows, were highly similar, despite stratifying by the individual drug used for IMT. Analysis stratified by cephapirin only (Figure 6B) more closely resembled that of the combination of cephapirin and cloxacillin (Figure 6A). Analysis stratified by cloxacillin only (Figure 6C) revealed similarities of the DRY and CTL samples with FRESH cows, independent of treatment. This similarity may have been driven by the aforementioned greater diversity in DRY sample time points in conjunction with a limited sample size, that may not had been sufficiently large to appropriately represent the abundance of the individual taxa. Together, these factors could have resulted in the lack of differentiation that we observed.

## 3. Discussion

### 3.1. Lack of Microbiota Differentiation of FRESH Samples from CTL and IMT Cows

No genera were deemed significant in differentiating FRESH samples from either CTL or IMT cows, indicating intramammary antimicrobial dry cow therapy had no significant effect on the udder milk microbiota post-partum (Figure 5B). A study by Derakhshani et al. (2018) [34] qualitatively evaluated the microbiota of the teat canal and mammary secretions of healthy udder quarters subjected to dry cow therapy using a long-acting antimicrobial product containing penicillin G and novobiocin, in combination with an internal teat sealant. Although shifts in the bacterial genera and phyla abundance were observed in their study, further analysis indicated a commonality between pre-IMT and postpartum microbiota of both teat canal and mammary secretions, indicating a limited effect of IMT on the microbiota. Another study by Ganda et al. (2016) [35] evaluated the impact of intramammary antimicrobial treatment on the milk microbiome of healthy cows and from cows presenting with clinical mastitis. Treatment with the third-generation cephalosporin ceftiofur had no significant effect on clinical cure, bacteriological cure, pathogen clearance, or bacterial load. Although this study focused on clinical cases of mastitis, they also observed similar results where antimicrobial drugs had little to no effect on milk microbiome and bacterial load. Our study did not include a third-generation cephalosporin treatment in the IMT group so our results are not directly comparable to this particular finding.

### 3.2. Microbiological Differentiation of DRY Samples from IMT and CTL Cows Compared to FRESH Samples

We observed that DRY cows, independent of treatment, had a greater microbial diversity when compared to milk from cows at FRESH (Figure 3). Similar findings for a greater diversity of milk microbiota at dry-off was also observed by Derakhshani et al. (2018). For both analyses evaluating microbial differences driving differentiation between time points by treatment, we only observed differentiated microbes in the DRY cow samples, independent of treatment (Figure 4). A possible explanation for this is that the cows in FRESH maintained a similar core microbiota as the DRY cows, but with a less diverse composition, leading to the observed difference in individual microbes in DRY samples.

The genera *Brevibacterium* and *Amaricoccus*, and the family Micrococcaceae were observed to have a significant canonical score for the discriminant analysis between the DRY samples from IMT cows (Figure 4A). Bacteria in the genus *Brevibacterium* are Gram positive, non-endospore forming, nonmotile, obligate aerobes, halotolerant, proteolytic, peptidolytic, esterolytic, and lipolytic in nature [36]. They have been isolated from human skin, marine, and terrestrial environments [37]. *Brevibacterium* is also found in the microbial communities present in raw milk and cheese [38]. A study on the microbial composition of Dutch “Danbo”, a surface ripened semi-hard cheese, revealed that, during ripening, *Brevibacterium* was the third most abundant genera on the cheese surface [39]. Research on udder cleft dermatitis found *Brevibacterium* was present in samples taken from three mild and one severe case, with 49.2% of classified reads belonging to *Brevibacterium* in the severe sample [40].

Although not reaching the ±0.3 canonical cut-off loading value, *Brevibacterium* was also observed for IMT discriminant analysis comparing CTL within DRY cows (Figure 5A). An explanation as to why *Brevibacterium* seems to be of greater relevance in the microbial composition of milk in DRY cows has yet to be determined.

The genus *Amaricoccus* was discovered in 1997 in activated sludge biomass from wastewater treatment plants around the world [41]. Bacteria in this genus are Gram negative aerobic cocci that can form tetrads, a grouping of four cells. *Amaricoccus* species are able to store polyhydroxyalkanoates (PHA), a biologically produced polymer similar to the plastics polyethylene and polypropylene [42]. Bacteria belonging to the genus may be able to degrade the antibacterial tricoslan [43]. *Amaricoccus* has also been detected in the microbiome of colostrum from human mothers who delivered via C-section [44]. It is uncertain why *Amaricoccus* was observed in great abundance in IMT cows.

The family Micrococcaceae includes Gram positive cocci bacteria found in dairy products and cured meats. Micrococcaceae includes the genus *Micrococcus* which, along with other members in the family, may reduce ripening times in cheese [45]. Micrococcaceae seem to be especially abundant in raw sheep milk cheeses, including the Spanish semi-soft “Casar de Cáceres” and soft “Tetilla” [46,47]. Micrococcaceae were more abundant on the surface of Tetilla cheese than the interior and may contribute to ripening [46]. Aside from dairy, Micrococcaceae are found in starter cultures for fermented meats and may prevent colonization of pathogenic bacteria by lowering the pH [48]. Micrococcaceae may also play a role in aroma development in the Spanish, fermented sausage “chorizo” [49]. It has also been detected in human breast milk of mothers living in mainland China and Taiwan, and in porcine breast milk samples collected from sows at various stages of pregnancy [50,51]. Given the locations where bacteria in the family Micrococcaceae have been found, it seems likely these bacteria are commensal milk bacteria.

### 3.3. Differentiated Bacteria from DRY and FRESH Samples Collected from CTL Cows

The genera *Akkermansia* and *Syntrophus* were observed to have a significant canonical score for the discriminant analysis between DRY and FRESH samples from CTL cows (Figure 4B). Bacteria in the genus *Akkermansia* are Gram-negative, obligate anaerobic, non-motile, and non-sporulating [52]. *Akkermansia muciniphila* plays an important role in the human gut microbiome as it is able to breakdown mucin in mucus as a carbon and nitrogen source [53]. Accounting for 1–4% of the bacteria in the adult intestine, *Akkermansia muciniphila* is inversely associated with diabetes, obesity, and other metabolic issues [54]. This health benefit suggests that *Akkermansia muciniphila* has potential as a future probiotic. Analysis of mice intestinal microbiota has also shown that *Akkermansia* growth can be affected by the consumption of cow and goat’s milk, as goat milk consumption had a positive effect on growth [55]. Although typically found in the intestine, the unique mucin-degrading ability of *Akkermansia muciniphila* may allow it to create a specific niche among the mammary gland microbiota. While the presence of *Akkermansia* in cow milk has not been previously reported, recent studies have detected the genus in human milk samples from subjects in Ghana and in the feces of lactating dairy cows [56,57]. 

Bacteria in the genus *Syntrophus* are Gram negative and strictly anaerobic. These bacteria get their energy from breaking down chemicals that might kill other bacteria such as phenol, benzoate, and fatty acids [58,59]. Bacteria in this genus are generally syntrophic, meaning they rely on partner organisms for key metabolites. *Syntrophus* has been identified in a study in which a 16s analysis of anaerobic digesters fed manure from cows was conducted [60]. The lack of data on *Syntrophus* in studies evaluating the microbiota of milk limit interpretation of the relevance of this bacterium. 

### 3.4. Differentiated Bacteria from DRY Samples Collected from IMT and CTL Cows

An unexpected finding of our study was that the milk microbiota of cows at DRY, sampled before the administration of treatment, differed in their microbial composition between the two treatment groups. As previously mentioned, a greater diversity of milk microbiota was observed for cows at DRY, and a greater individual milk microbiota diversity could have resulted in the observed findings. Nevertheless, only two genera were observed to significantly discriminate between DRY sampling points when comparing CTL and IMT, namely the genus *Alkalibacterium* for DRY samples from IMT cows, and the genus *Mogibacterium* for DRY samples from CTL cows (Figure 5A). Bacteria in the genus *Alkalibacterium* are Gram positive, non-sporulating, and found in various basic environments [61]. In addition to the previously mentioned *Brevibacterium*, *Alkalibacterium* can also be found on the surface of Dutch “Danbo” cheese—accounting for about 1.3% of total ASVs [39]. It was hypothesized that *Alkalibacterium* was introduced to the cheese via the sea salt used in brining. However, given the presence of *Alkalibacterium* in our milk samples, it is possible the bacteria were instead selected for by the saline brine used in the cheese making process. It was also found on the rind of a blue-veined, raw milk cheese from the UK made in the style of blue stilton cheese [62]. In this case, it is believed that the alkaline pH found in the mature rind could have selected for *Alkalibacterium.* Aside from cheese, *Alkalibacterium* has also been found in the fermentation of Spanish-style green table olives and indigo dye [63,64]. It has also been discovered to have the potential to recover up to 52% of the copper present in waste produced by the burning of solid waste via a process called bioleaching [65]. Lastly, the species *Alkalibacterium kapii* has been found to inhibit the growth of *Listeria* when present on the surface of cheese [66].

Bacteria in the genus *Mogibacterium* are Gram positive, non-spore forming, obligate anaerobes. Interestingly, research has shown that *Mogibacterium* are significantly more abundant in the rumen of high-methane producing cattle [67]. *Mogibacterium* has also been shown to decrease in abundance in dairy cows fed a high-grain diet designed to induce subacute ruminal acidosis [68]. The authors concluded that use of a high-grain diet may increase the risk of mastitis.

## 4. Material and Methods

### 4.1. Sampling

#### Milk Samples

A random subset of 100 milk samples from 50 cows on 3 of the 8 study herds in the original trial described below were selected [24]. Choice of the 3 herds was based on the geographic representation of herds from California’s San Joaquin Valley and to minimize the number of freeze–thaw cycles. Specifically, the three herds were in Stanislaus (1600 milking Holsteins; bulk tank somatic cell count (BTSCC) 200,000 cells/mL), San Joaquin (1800 milking Holsteins; BTSCC 145,000 cells/mL), and Tulare counties (1100 milking Jerseys; BTSCC 250,000 cells/mL). At dry-off, no cows with clinical signs of mastitis, health events, body condition score <2.5, lameness, or non-functional quarters were enrolled. Cows were randomized to one of four groups at dry-off and received either intramammary infusion (IMT), internal teat sealant, or both. For the current study, the 50 cows were randomly selected from cows that either did not receive any treatment at dry-off or received only intramammary antibiotic infusion. The decision to utilize the dry-off (DRY) and post calving (FRESH) milk samples from each of the 50 randomly selected cows, which yielded 100 milk samples, was based on budgetary reasons.

The decision to use cephapirin or cloxacillin was at the farm level, with 2 farms enrolled in the study using cephapirin and one farm using cloxacillin. Of the 50 cows, 12 from the San Joaquin herd and 4 from the Tulare herd received intramammary infusion (IMT) with cephapirin benzathine (ToMORROW^®^, Boehringer Ingelheim Animal Health USA Inc., Duluth, GA, USA) and 7 from the Stanislaus herd received cloxacillin benzathine (Dry-Clox^®^, Boehringer Ingelheim Animal Health USA Inc., Duluth, GA, USA) at the time of dry-off following the manufacturer’s label instructions. The remaining 27 cows from San Joaquin (*n* = 13), Tulare (*n* = 8) and Stanislaus (*n* = 6) received no intramammary infusion (CTL) at the time of dry-off. 

### 4.2. DNA Extraction 

DNA was extracted using a standardized approach [25]. A total of 6 mL of thawed milk from each sample was centrifuged at 8500 rpm for 5 min in a sterile 15 mL conical centrifuge tube to pellet bacteria. Whey and fat were then discarded. A total of 500 µL of PowerTube buffer was added to conical tube and then vortexed briefly to loosen the pellet. A 10-min incubation at 65 °C of the sample then followed to increase DNA output. DNA extraction then continued as recommended by the DNeasy PowerSoil Kit (Qiagen N.V., Carlsbad, CA, USA). A total of 100 µL of DNA was eluted for each sample into a sterile 2 mL micro centrifuge tube. DNA samples were stored at −80 °C until further processing.

### 4.3. PCR Amplification of 16S rRNA and DNA Sequencing 

Due to the small quantities of DNA present in each sample, an additional step was required to amplify DNA to a necessary concentration for 16s sequencing [26]. In this additional step, primers 27F-YM+4 and 1492R were used to amplify the nearly full length 16s rRNA. The 27F-YM+4 primer mix is an eightfold-degenerate primer containing four parts 27F-YM (AGAGTTTGATYMTGGCTCAG), plus one part each of primers specific for the amplification of *Atopobium* (AGAGTTCGATCCTGGCTCAG), *Bifidobacteriaceae* (AGGGTTCGATTCTGGCTCAG), *Borrelia* (AGAGTTTGATCCTGGCTTAG), and *Chlamydiales* (AGAATTTGATCTTGGTTCAG). Each 25 μL PCR reaction contained 1 Unit Kapa2G Robust Hot Start Polymerase (Kapa Biosystems, Inc., Wilmington, MA, USA), 1.5 mM MgCl_2_, 0.2 mM final concentration dNTP mix, 0.2 μM final concentration of each primer, and 2 μL of DNA for each sample. PCR conditions were: an initial incubation at 95 °C for 3 min, followed by 25 cycles of 95 °C for 45 s, 50 °C for 30 s, 72 °C for 30 s, and a final extension of 72 °C for 3 min.

Following amplification of the full length 16s rRNA, primers 319F and 806R were used to specifically amplify the V3-V4 domain of the 16S rRNA. Each 25 μL PCR reaction contained 1 Unit Kapa2G Robust Hot Start Polymerase (Kapa Biosystems, Inc., Wilmington, MA, USA), 1.5 mM MgCl_2_, 0.2 mM final concentration dNTP mix, 0.2 μM final concentration of each primer, and 1 μL of DNA for each sample. An initial incubation at 95 °C for 3 min, followed by 25 cycles of 95 °C for 45 s, 50 °C for 30 s, 72 °C for 30 s, and a final extension of 72 °C for 3 min comprised the PCR conditions.

In the final PCR run, each sample was barcoded with a unique forward and reverse barcode combination. The PCR reaction in step three contained 1 Unit Kapa2G Robust Hot Start Polymerase (Kapa Biosystems, Inc., Wilmington, MA, USA), 1.5 mM MgCl_2_, 0.2 mM final concentration dNTP mix, 0.2 μM final concentration of each uniquely barcoded primer, and 1 ul of the product from the PCR reaction in step two. PCR conditions were: an initial incubation at 95 °C for 3 min, followed by 8 cycles of 95 °C for 30 s, 58 °C for 30 s, 72 °C for 30 s, and a final extension of 72 °C for 3 min. The product of this final PCR reaction was quantified on the Qubit instrument using the Qubit Broad Range DNA kit (Invitrogen/Life Technologies Inc., Carlsbad, CA, USA).

Individual amplicons were pooled in equal concentrations and the pooled library was cleaned utilizing Ampure XP beads (Beckman Coulter Life Sciences, Indianapolis, IN, USA). The library was quantified via qPCR followed by 300-bp paired-end sequencing using an Illumina MiSeq platform. Forward and reverse reads were trimmed to 260 bp and 200 bp, respectively, before proceeding with the DADA2 portion of the QIIME2 analysis pipeline.

### 4.4. Bioinformatics

The 16S rRNA sequencing data was demultiplexed with dbcAmplicons (Matt Settles, UC Davis Bioinformatics Core Facility, Davis, CA, USA) and processed through the Quantitative Insights into Microbial Ecology 2 (QIIME2) version 2018.6 utilizing the DADA2 pipeline [27,28]. Taxonomy was assigned using Greengenes version 13.8 at a 99% match [29]. All sample libraries were rarefied at an equal depth of 16,000 reads using QIIME2 prior to generating Shannon diversity indices.

### 4.5. Statistical Analysis

Descriptive data was analyzed using JMP Pro 14.0. Distribution of amplicon sequence variants (ASVs) in milk samples from cows by treatment group and time point were visualized using a Venn diagram. Shannon diversity was displayed using a quantile-box plot.

Relative abundances of different bacterial taxa in each sample were used as covariates in the stepwise discriminant analysis models built in JMP Pro 14.0. Each variable was removed in a stepwise manner until only variables with a *p* value  <  0.05 were retained in the final model. Groups used in the analysis were the treatment group and sampling time point combinations. Analyses were conducted, both independent of the specific antimicrobial treatment being used, as well as by stratifying the dataset according to the antibiotic treatment and the interactions between treatment and time points. Two separate models were built to evaluate bacterial taxa. The first model did not consider the type of antimicrobial used at dry-off. In the second model, data were stratified by antimicrobial treatment (cephapirin and cloxacillin). For models not differentiating between the antibiotics used for IMT, canonical values for these analyses were used to create a graphical display of the taxonomical results. A canonical cut-off value of ±0.3 was used.

For taxa identified as having a significant effect on the microbial composition for each treatment group and time interaction, a linear regression was used to evaluate the potential significant differences in relative abundance for the taxa of interest. Multilinear regression models were generated, where the relative abundance for each taxon with a canonical value of ±0.3 was used as the dependent variable, and the treatment groups, time points, and interaction were included as explanatory variables. For each model, the animal individual identifier was nested within the farm where the cow was located, and inserted in the model as a random effect. If the time and treatment interaction between the sampling time points and treatment groups were significant, a pairwise comparison analysis was conducted using Tukey’s Honest Significant Difference (HSD) [30]. Differences were considered significant when a *p* value < 0.05 was observed.

Relative mean abundances for six genera associated with mastitis (*Staphylococcus*, *Bacillus*, *Streptococcus*, *Mycoplasma*, *Escherichia*, and *Trueperella*) were compared at DRY and FRESH time points for both IMT and CTL (Supplementary Figure S1). For this analysis a linear regression was used, where the dependent variable was the bacteria genus relative abundance, and the independent variable was the time point and treatment group variable, as well as its interaction. The individual identifier of the cow was nested by the farm and it was sampled and entered in the model as a random effect.

## 5. Conclusions

IMT group samples collected at dry-off had a greater correlation with the genera *Brevibacterium* and *Amaricoccus*, and the family Micrococcaceae, when compared to CTL for the FRESH samples. Furthermore, CTL group samples collected at DRY had a greater correlation with the genera *Akkermansia* and *Syntrophus,* when compared to FRESH samples. For DRY samples collected from the IMT group, a greater correlation for the genus *Alkalibacterium* was observed when compared to CTL samples. Future research to evaluate the impacts of the findings related to the prevalence of different taxa on individual animal health and production are needed. No correlations between taxa were observed for FRESH samples, comparing CTL and IMT treatment groups. Taken together, the lack of genera deemed significant in differentiating FRESH samples from either CTL or IMT cows, indicated intramammary antimicrobial dry cow therapy based on the drugs used in our study had no significant effect on the udder milk microbiota post-partum.

## Figures and Tables

**Table 1:** Relative abundances for the top 20 taxa observed by treatment group and sampling point.

TAXON ^3^	Treatment/Time, % (SE)			
	CTL ^1^		IMT ^2^	
	DRY ^4^	FRESH ^5^	DRY ^4^	FRESH
g__Staphylococcus	21.3 (4.7)	19.2 (5.6)	13.7 (3.7)	15.7 (3.9)
g__Delftia	11.4 (3.8)	19.7 (5.2)	19.9 (6.3)	20 (5)
f__Peptostreptococcaceae	7.6 (1.7)	5.1 (1.2)	6.2 (0.9)	3.0 (1.0)
f__Ruminococcaceae	4.8 (1.2)	5.3 (1.2)	5.7 (1.8)	4.7 (1.3)
g__Corynebacterium	3.9 (1.3)	2.2 (0.9)	3.1 (1.2)	2.5 (0.5)
g__Turicibacter	3.3 (0.8)	1.9 (0.6)	2.8 (0.6)	3.3 (1.2)
o__Clostridiales	2.7 (0.6)	1.2 (0.3)	3.1 (0.9)	1.8 (0.6)
f__Lachnospiraceae	2.6 (0.6)	1.4 (0.4)	1.7 (0.6)	2.1 (0.7)
g__Serratia	0.7 (0.7)	2.5 (2.5)	1.6 (1.6)	1.5 (1.5)
o__Bacteroidales	1.1 (0.4)	1.2 (0.5)	1.2 (0.5)	2.3 (1.4)
g__Epulopiscium	0.6 (3.4)	4 (3.6)	0.1 (0)	0.1 (0.1)
g__Streptococcus	1 (0.4)	1.2 (0.9)	0.7 (0.3)	2.2 (2.1)
g__Acinetobacter	1.3 (1.0)	0.8 (0.5)	1.1 (0.4)	1.7 (0.6)
f__Clostridiaceae	1.5 (0.4)	0.9 (0.3)	1 (0.3)	1.3 (0.6)
g__5-7N15	1.3 (0.5)	1.1 (0.4)	1.2 (0.5)	1.0 (3.7)
g__Salinicoccus	1.1 (0.6)	0.4 (0.2)	1.4 (0.6)	1.7 (1.2)
g__Herbaspirillum	0.7 (0.6)	0.4 (0.2)	0 (0)	3.5 (2.4)
f__Neisseriaceae	0.9 (0.5)	1.0 (0.7)	1.1 (7.6)	0.8 (0.7)
g__Bacillus	0.8 (0.3)	0.7 (0.3)	1.2 (0.4)	0.8 (0.5)
f__Aerococcaceae	0.5 (0.2)	0.5 (0.2)	1.2 (0.4)	1.2 (0.7)

^1^ CTL: control group representing cows not receiving intramammary antimicrobial drug treatment at dry-off; ^2^ IMT: treatment group representing cows receiving intramammary antimicrobial drug treatment at dry-off; ^3^ Taxon: refers to the various levels of classification used to describe sequence data (g—genus, f—family, and o—order); ^4^ DRY: samples collected at the time of dry-off; ^5^ FRESH samples: samples collected from post-partum cows between 4 and 11 days in milk.

## Data Availability

The de-identified data set was made available to reviewers, and is available on request due to privacy restrictions.

## Supplementary Materials

The following supporting information can be downloaded at: https://www.mdpi.com/article/10.3390/antibiotics11070963/s1, Figure S1: Relative mean abundance of the genus *Staphylococcus* sp., *Bacillus* sp., *Streptococcus* sp., *Mycoplasma* sp., *Escherichia* sp., and *Trueperella* sp. by treatment group (CTL and IMT) for both DRY and FRESH time points. Error bars correspond to a 95% confidence interval; Table S1: Results from multivariate models for each taxon with a canonical value of ±0.3 for analysis in Figures 2 and 3. Results are only displayed for the interaction variable between sampling time points and the treatment group; Table S2: Results from Tukey pairwise analysis for the genus *Brevibacterium* comparing all treatment groups and sampling time point interactions.

## References

[1] Cha E., Kristensen A.R., Hertl J., Schukken Y., Tauer L., Welcome F.L., Gröhn Y. (2014). Optimal insemination and replacement decisions to minimize the cost of pathogen-specific clinical mastitis in dairy cows. J. Dairy Sci..

[2] Ruegg P.L. (2017). A 100-Year Review: Mastitis detection, management, and prevention. J. Dairy Sci..

[3] Hudson C.D., Bradley A.J., Breen J.E., Green M.J. (2012). Associations between udder health and reproductive performance in United Kingdom dairy cows. J. Dairy Sci..

[4] Fuenzalida M.J., Fricke P.M., Ruegg P.L. (2015). The association between occurrence and severity of subclinical and clinical mastitis on pregnancies per artificial insemination at first service of Holstein cows. J. Dairy Sci..

[5] USDA (2016). Dairy 2014, Milk Quality, Milking Procedures, and Mastitisin the United States, 2014.

[6] FDA (2021). 2020 Summary Report on Antimicrobials Soldor Distributed for Usein Food-Producing Animals.

[7] WHO (2019). Critically Important Antimicrobials for Human Medicine.

[8] National Mastitis Council (2017). Current Concepts of Bovine Mastitis.

[9] Ward G.E., Schultz L.H. (1974). Incidence and Control of Mastitis During the Dry Period. J. Dairy Sci..

[10] Rindsig R.B., Rodewald R.G., Smith A., Spahr S. (1978). Complete Versus Selective Dry Cow Therapy for Mastitis Control. J. Dairy Sci..

[11] Woolford M.W., Williamson J.H., Day A.M., Copeman P.J. (1998). The prophylactic effect of a teat sealer on bovine mastitis during the dry period and the following lactation. N. Z. Vet.-J..

[12] Bonsaglia E.C., Gomes M.S., Canisso I.F., Zhou Z., Lima S.F., Rall V.L.M., Oikonomou G., Bicalho R.C., Lima F.S., Bonsaglia E.C. (2017). Milk microbiome and bacterial load following dry cow therapy without antibiotics in dairy cows with healthy mammary gland. Sci. Rep..

[13] Huijps K., Hogeveen H. (2007). Stochastic Modeling to Determine the Economic Effects of Blanket, Selective, and No Dry Cow Therapy. J. Dairy Sci..

[14] Scherpenzeel C.G.M., Hogeveen H., Maas L., Lam T. (2018). Economic optimization of selective dry cow treatment. J. Dairy Sci..

[15] Vasquez A.K., Nydam D.V., Foditsch C., Wieland M., Lynch R., Eicker S., Virkler P. (2018). Use of a culture-independent on-farm algorithm to guide the use of selective dry-cow antibiotic therapy. J. Dairy Sci..

[16] Oikonomou G., Machado V.S., Santisteban C., Schukken Y., Bicalho R.C. (2012). Microbial Diversity of Bovine Mastitic Milk as Described by Pyrosequencing of Metagenomic 16s rDNA. PLoS ONE.

[17] Kuehn J.S., Gorden P., Munro D., Rong R., Dong Q., Plummer P.J., Wang C., Phillips G.J. (2013). Bacterial Community Profiling of Milk Samples as a Means to Understand Culture-Negative Bovine Clinical Mastitis. PLoS ONE.

[18] Metzger S.A., Hernandez L.L., Skarlupka J., Walker T.M., Suen G., Ruegg P.L. (2018). A Cohort Study of the Milk Microbiota of Healthy and Inflamed Bovine Mammary Glands from Dryoff Through 150 Days in Milk. Front. Vet.-Sci..

[19] Ganda E.K., Gaeta N.C., Sipka A., Pomeroy B., Oikonomou G., Schukken Y., Bicalho R.C. (2017). Normal milk microbiome is reestablished following experimental infection with Escherichia coli independent of intramammary antibiotic treatment with a third-generation cephalosporin in bovines. Microbiome.

[20] Belkaid Y., Hand T.W. (2014). Role of the Microbiota in Immunity and Inflammation. Cell.

[21] Olsan E.E., Byndloss M., Faber F., Rivera-Chávez F., Tsolis R.M., Bäumler A.J. (2017). Olonization resistance: The deconvolution of a complex trait. J. Biol. Chem..

[22] Keeney K.M., Yurist-Doutsch S., Arrieta M.-C., Finlay B.B. (2014). Effects of Antibiotics on Human Microbiota and Subsequent Disease. Annu. Rev. Microbiol..

[23] Derakhshani H., Fehr K.B., Sepehri S., Francoz D., De Buck J., Barkema H., Plaizier J.C., Khafipour E. (2018). Invited review: Microbiota of the bovine udder: Contributing factors and potential implications for udder health and mastitis susceptibility. J. Dairy Sci..

[24] Aly S.S., Okello E., ElAshmawy W.R., Williams D.R., Anderson R.J., Rossitto P., Tonooka K., Glenn K., Karle B., Lehenbauer T.W. (2022). Effectiveness of Intramammary Antibiotics, Internal Teat Sealants, or Both at Dry-Off in Dairy Cows: Clinical Mastitis and Culling Outcomes. Antibiotics.

[25] Lima S.F., Bicalho M.L.S., Bicalho R.C. (2018). Evaluation of milk sample fractions for characterization of milk microbiota from healthy and clinical mastitis cows. PLoS ONE.

[26] Frank J.A., Reich C.I., Sharma S., Weisbaum J.S., Wilson B.A., Olsen G.J. (2008). Critical Evaluation of Two Primers Commonly Used for Amplification of Bacterial 16S rRNA Genes. Appl. Environ. Microbiol..

[27] Callahan B.J., Mcmurdie P.J., Rosen M.J., Han A.W., Johnson A.J.A., Holmes S.P. (2016). DADA2: High-resolution sample inference from Illumina amplicon data. Nat. Methods.

[28] Bolyen E., Rideout J.R., Dillon M.R., Bokulich N.A., Abnet C., Al-Ghalith G.A., Alexander H., Alm E.J., Arumugam M., Asnicar F. (2019). QIIME2: Reproducible, interactive, scalable, and extensible microbiome data science. Nat. Biotechnol..

[29] McDonald D., Price M.N., Goodrich J., Nawrocki E.P., DeSantis T.Z., Probst A., Andersen G.L., Knight R., Hugenholtz P. (2012). An improved Greengenes taxonomy with explicit ranks for ecological and evolutionary analyses of bacteria and archaea. ISME J..

[30] Midway S., Robertson M., Flinn S., Kaller M. (2020). Comparing multiple comparisons: Practical guidance for choosing the best multiple comparisons test. PeerJ.

[31] Lambert Z.V., Durand R.M. (1975). Some precautions in using canonical analysis. J. Mark. Res..

[32] Jeon S.J., Neto A.V., Gobikrushanth M., Daetz R., Mingoti R.D., Parize A.C.B., de Freitas S.L., da Costa A.N.L., Bicalho R.C., Lima S. (2015). Uterine Microbiota Progression from Calving until Establishment of Metritis in Dairy Cows. Appl. Environ. Microbiol..

[33] Pandey P., Chiu C., Miao M., Wang Y., Settles M., Del Rio N.S., Castillo A., Souza A., Pereira R., Jeannotte R. (2018). 16S rRNA analysis of diversity of manure microbial community in dairy farm environment. PLoS ONE.

[34] Derakhshani H., Plaizier J.C., De Buck J., Barkema H., Khafipour E. (2018). Composition of the teat canal and intramammary microbiota of dairy cows subjected to antimicrobial dry cow therapy and internal teat sealant. J. Dairy Sci..

[35] Ganda E.K., Bisinotto R.S., Lima S.F., Kronauer K., Decter D.H., Oikonomou G., Schukken Y., Bicalho R.C. (2016). Longitudinal metagenomic profiling of bovine milk to assess the impact of intramammary treatment using a third-generation cephalosporin. Sci. Rep..

[36] Rattray F.P., Fox P.F. (1999). Aspects of Enzymology and Biochemical Properties of Brevibacterium linens Relevant to Cheese Ripening: A Review. J. Dairy Sci..

[37] Ivanova E.P., Christen R., Alexeeva Y.V., Zhukova N., Gorshkova N.M., Lysenko A.M., Mikhailov V.V., Nicolau D.V. (2004). Brevibacterium celere sp. nov., isolated from degraded thallus of a brown alga. Int. J. Syst. Evol. Microbiol..

[38] Bal Z.S., Sen S., Karapinar D.Y., Aydemir S., Vardar F. (2015). The first reported catheter-related *Brevibacterium casei* bloodstream infection in a child with acute leukemia and review of the literature. Braz. J. Infect. Dis..

[39] Ryssel M., Johansen P., Abu Al-Soud W., Sørensen S., Arneborg N., Jespersen L. (2015). Microbial diversity and dynamics throughout manufacturing and ripening of surface ripened semi-hard Danish Danbo cheeses investigated by culture-independent techniques. Int. J. Food Microbiol..

[40] Ekman L., Bagge E., Nyman A., Waller K.P., Pringle M., Segerman B. (2020). A shotgun metagenomic investigation of the microbiota of udder cleft dermatitis in comparison to healthy skin in dairy cows. PLoS ONE.

[41] Maszenan A.M., Seviour R.J., Patel B., Rees G., McDougall B.M. (1997). *Amaricoccus* gen. nov., a Gram-Negative Coccus Occurring in Regular Packages or Tetrads, Isolated from Activated Sludge Biomass, and Descriptions of *Amaricoccus veronensis* sp. nov., *Amaricoccus tamworthensis* sp. nov., *Amaricoccus macauensis* sp. nov., and *Amaricoccus kaplicensis* sp. nov. Int. J. Syst. Bacteriol..

[42] Puyol D., Batstone D.J., Hülsen T., Astals S., Peces M., Krömer J. (2017). Resource Recovery from Wastewater by Biological Technologies: Opportunities, Challenges, and Prospects. Front. Microbiol..

[43] Jia J.-X., Gao J.-F., Dai H.-H., Zhang W.-Z., Zhang D., Wang Z.-Q. (2020). DNA-based stable isotope probing identifies triclosan degraders in nitrification systems under different surfactants. Bioresour. Technol..

[44] Toscano M., De Grandi R., Peroni D.G., Grossi E., Facchin V., Comberiati P., Drago L. (2017). Impact of delivery mode on the colostrum microbiota composition. BMC Microbiol..

[45] Buffa M., Guamis B., Royo C., Trujillo A.-J. (2001). Microbiological changes throughout ripening of goat cheese made from raw, pasteurized and high-pressure-treated milk. Food Microbiol..

[46] Menéndez S., Godínez R., Centeno J., Rodríguez-Otero J. (2001). Microbiological, chemical and biochemical characteristics of ‘Tetilla’ raw cows-milk cheese. Food Microbiol..

[47] Cáceres P., Castillo D., Pizarro M. (1997). Secondary flora of Casar de Cáceres cheese: Characterization of Micrococcaceae. Int. Dairy J..

[48] Montel M.-C., Reitz J., Talon R., Berdagué J.-L., Rousset-Akrim S. (1996). Biochemical activities of Micrococcaceae and their effects on the aromatic profiles and odours of a dry sausage model. Food Microbiol..

[49] Garcia-Varona M., Santos E.M., Jaime I., Rovira J. (2000). Characterisation of Micrococcaceae isolated from different varieties of chorizo. Int. J. Food Microbiol..

[50] Chen W., Mi J., Lv N., Gao J., Cheng J., Wu R., Ma J., Lan T., Liao X. (2018). Lactation Stage-Dependency of the Sow Milk Microbiota. Front. Microbiol..

[51] Li S.-W., Watanabe K., Hsu C.-C., Chao S.-H., Yang Z.-H., Lin Y.-J., Chen C.-C., Cao Y.-M., Huang H.-C., Chang C.-H. (2017). Bacterial Composition and Diversity in Breast Milk Samples from Mothers Living in Taiwan and Mainland China. Front. Microbiol..

[52] Naito Y., Uchiyama K., Takagi T. (2018). A next-generation beneficial microbe: *Akkermansia muciniphila*. J. Clin. Biochem. Nutr..

[53] Belzer C., De Vos W.M. (2012). Microbes inside—From diversity to function: The case of Akkermansia. ISME J..

[54] Cani P.D., De Vos W.M. (2017). Next-Generation Beneficial Microbes: The Case of *Akkermansia muciniphila*. Front. Microbiol..

[55] Wang Z., Jiang S., Ma C., Huo D., Peng Q., Shao Y., Zhang J. (2018). Evaluation of the nutrition and function of cow and goat milk based on intestinal microbiota by metagenomic analysis. Food Funct..

[56] Lackey K.A., Williams J.E., Meehan C.L., Zachek J.A., Benda E.D., Price W.J., Foster J.A., Sellen D.W., Kamau-Mbuthia E.W., Kamundia E.W. (2019). What’s Normal? Microbiomes in Human Milk and Infant Feces Are Related to Each Other but Vary Geographically: The INSPIRE Study. Front. Nutr..

[57] Huang S., Ji S., Yan H., Hao Y., Zhang J., Wang Y., Cao Z., Li S. (2020). The day-to-day stability of the ruminal and fecal microbiota in lactating dairy cows. Microbiol. Open.

[58] De Godoi L.A.G., Fuess L.T., Delforno T.P., Foresti E., Damianovic M. (2018). Characterizing phenol-removing consortia under methanogenic and sulfate-reducing conditions: Potential metabolic pathways. Environ. Technol..

[59] McInerney M.J., Rohlin L., Mouttaki H., Kim U., Krupp R.S., Rios-Hernandez L., Sieber J., Struchtemeyer C.G., Bhattacharyya A., Campbell J.W. (2007). The genome of *Syntrophus aciditrophicus*: Life at the thermodynamic limit of microbial growth. Proc. Natl. Acad. Sci. USA.

[60] Spirito C.M., Daly S.E., Werner J.J., Angenent L.T. (2018). Redundancy in Anaerobic Digestion Microbiomes during Disturbances by the Antibiotic Monensin. Appl. Environ. Microbiol..

[61] Ishikawa M., Tanasupawat S., Nakajima K., Kanamori H., Ishizaki S., Kodama K., Okamoto-Kainuma A., Koizumi Y., Yamamoto Y., Yamasato K. (2009). *Alkalibacterium thalassium* sp. nov., *Alkalibacterium pelagium* sp. nov., *Alkalibacterium putridalgicola* sp. nov. and *Alkalibacterium kapii* sp. nov., slightly halophilic and alkaliphilic marine lactic acid bacteria isolated from marine organisms and salted foods collected in Japan and Thailand. Int. J. Syst. Evol. Microbiol..

[62] Yunita D., Dodd C.E. (2018). Microbial community dynamics of a blue-veined raw milk cheese from the United Kingdom. J. Dairy Sci..

[63] Lucena-Padrós H., Jiménez E., Maldonado-Barragán A., Rodríguez J.M., Ruiz-Barba J.L. (2015). PCR-DGGE assessment of the bacterial diversity in Spanish-style green table-olive fermentations. Int. J. Food Microbiol..

[64] Okamoto T., Aino K., Narihiro T., Matsuyama H., Yumoto I. (2017). Analysis of microbiota involved in the aged natural fermentation of indigo. World J. Microbiol. Biotechnol..

[65] Ramanathan T., Ting Y.-P. (2016). Alkaline bioleaching of municipal solid waste incineration fly ash by autochthonous extremophiles. Chemosphere.

[66] Roth E., Schwenninger S.M., Eugster-Meier E., Lacroix C. (2011). Facultative anaerobic halophilic and alkaliphilic bacteria isolated from a natural smear ecosystem inhibit Listeria growth in early ripening stages. Int. J. Food Microbiol..

[67] Wallace R.J., Rooke J.A., McKain N., Duthie C.-A., Hyslop J.J., Ross D.W., Waterhouse A., Watson M., Roehe R. (2015). The rumen microbial metagenome associated with high methane production in cattle. BMC Genom..

[68] Zhang R., Huo W., Zhu W., Mao S. (2014). Characterization of bacterial community of raw milk from dairy cows during subacute ruminal acidosis challenge by high-throughput sequencing. J. Sci. Food Agric..

