# Supplementary material for: Effect of Intramammary Dry Cow Antimicrobial Treatment on Fresh Cow’s Milk Microbiota in California Commercial Dairies
Source: Antibiotics (Basel). 2022 Jul 18;11(7):963. doi: 10.3390/antibiotics11070963 (PMC9312063; doi:10.3390/antibiotics11070963)
Supplement: Supplementary file 1 [file antibiotics-11-00963-s001.zip › antibiotics-1808343-supplementary.pdf]

Supplemental Figure S1. Relative mean abundance of the genus *Staphylococcus sp*, *Bacillus sp* , *Streptococcus sp*, *Mycoplasma sp*, *Escherichia sp*, and *Trueperella sp* by treatment group (CTL and IMT) for both DRY and FRESH time points. Error bars correspond to 95% confidence interval.

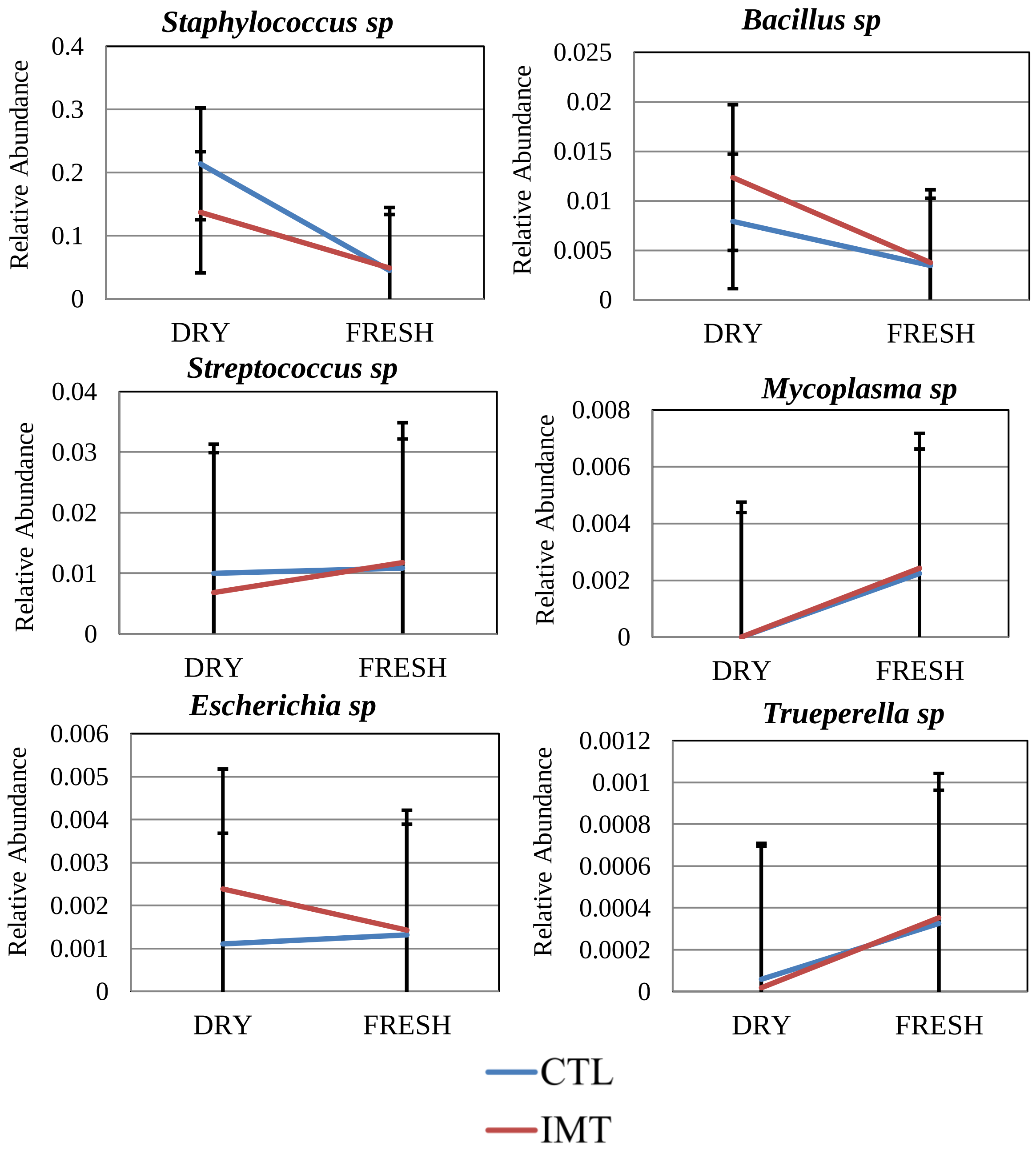

Supplemental Table S1. Results from multivariate models for each taxon with a canonical value of ± 0.3 for analysis in figures 2 and 3. Results are only displayed for the interaction variable between sampling time points and treatment group.

**Taxon[1]**	**Estimate[2]**	**Std Error[3]**	***P* value[4]**
**g_syntrophus**	-0.001253	0.001517	0.4127
**f_micrococcaceae**	-0.000253	0.000384	0.5135
**g_amaricoccus**	-0.000594	0.000523	0.2618
**g_Proprionibacterium**	0.0005021	0.000587	0.3963
**g_pseudomonas**	-0.003282	0.00187	0.0857
**g_akkermansia**	2.21E-06	8.49E-05	0.9793
**g_brevibacterium**	-0.000656	0.000294	**0.0303**

1. Taxon: refers to the various levels of classification used to describe sequence data (g-genus and f-family)
2. Parameter estimate for the multivariate model evaluating interaction between sampling time points and treatment group.
3. Standard error for the model estimate for the variable representing interaction between treatment group and sampling time point
4. *P*-value for the variable representing interaction between treatment groups and sampling time point. A *P*-value < 0.05 indicates that at least one sampling time and treatment interactions was significantly different.

Supplemental Table S2. Results from Tukey pairwise analysis for the genus *Brevibacterium* comparing all treatment groups and sampling time point interactions.

**Groups compared** [1]		**Difference**[2]	**Std Error**[3]	***P* value**[4]
**DRY-CTL**	**DRY-IMT**	-0.001463	0.0008194	0.2927
**DRY-CTL**	**FRESH-CTL**	-0.000797	0.000797	0.75
**DRY-CTL**	**FRESH-IMT**	0.000363	0.0008194	0.9708
**DRY-IMT**	**FRESH-CTL**	0.000666	0.0008194	0.8482
**DRY-IMT**	**FRESH-IMT**	0.001826	0.0008635	0.1632
**FRESH-CTL**	**FRESH-IMT**	0.00116	0.0008194	0.4962

1. Group interactions (treatment group and sampling time point) compared using Tukey pairwise analysis.
2. Absolute difference in the means between groups compared as determined by Tukey pairwise analysis.
3. Standard error for difference in the means
4. *P* value for the variable representing a significant difference (P-value < 0.05) between the means of the two groups being compared using Tukey pairwise analysis.
